# Multiscale information processing in the immune system

**DOI:** 10.3389/fimmu.2025.1563992

**Published:** 2025-07-21

**Authors:** Roberto Navarro Quiroz, Jose Villarreal Camacho, Eloina Zarate Peñata, Yesit Bello Lemus, Claudio López-Fernández, Lorena Gomez Escorcia, Cecilia Fernández-Ponce, Martha Rebolledo Cobos, Jennifer Fandiño Moreno, Ornella Fiorillo-Moreno, Elkin Navarro Quiroz

**Affiliations:** ^1^ Center for Research in Critical Dynamics, Barranquilla, Colombia; ^2^ Faculty of Medicine, Universidad Libre, Barranquilla, Colombia; ^3^ Universidad Metropolitana, Barranquilla, Colombia; ^4^ Faculty of Basic and Biomedical Sciences, Center for Research in Life Sciences, Universidad Simón Bolívar, Barranquilla, Colombia; ^5^ Faculty of Physical and Mathematical Sciences, University of Chile, Santiago de Chile, Chile; ^6^ Department of Biomedicine, Biotechnology and Public Health, University of Cadiz, Cadiz, Spain; ^7^ Institute of Biomedical Research Cadiz (INIBICA), Cadiz, Spain; ^8^ Fundación Universitaria San Martín, Puerto Colombia, Colombia; ^9^ Clínica Iberoamericana, Barranquilla, Colombia; ^10^ Clínica El Carmen, Barranquilla, Colombia

**Keywords:** multiscale information processing, adaptive immune networks, criticality and antifragility, canonical processing functions, systems immunology

## Abstract

The immune system is an advanced, multiscale adaptive network capable of processing biological information across molecular, cellular, tissue, and systemic levels, demonstrating remarkable properties such as antifragility and criticality. We propose a unified theoretical framework based on six canonical functions—sensing, coding, decoding, response, feedback, and learning—that act as scale-invariant operational units, integrating molecular precision, collective cellular intelligence, and systemic coordination into coherent adaptive responses. Through this lens, immune function emerges from universal principles of complex network organization, including symmetry breaking, self-organized criticality, modularity, and small-world topology. These insights pave the way toward a predictive immunology grounded in fundamental physical principles, enabling novel computational modeling approaches and facilitating personalized therapeutic interventions that exploit inherent immunological robustness and plasticity.

## 1 Introduction

### 1.1 The immune system as an adaptive information processing network

**Figure 1:**
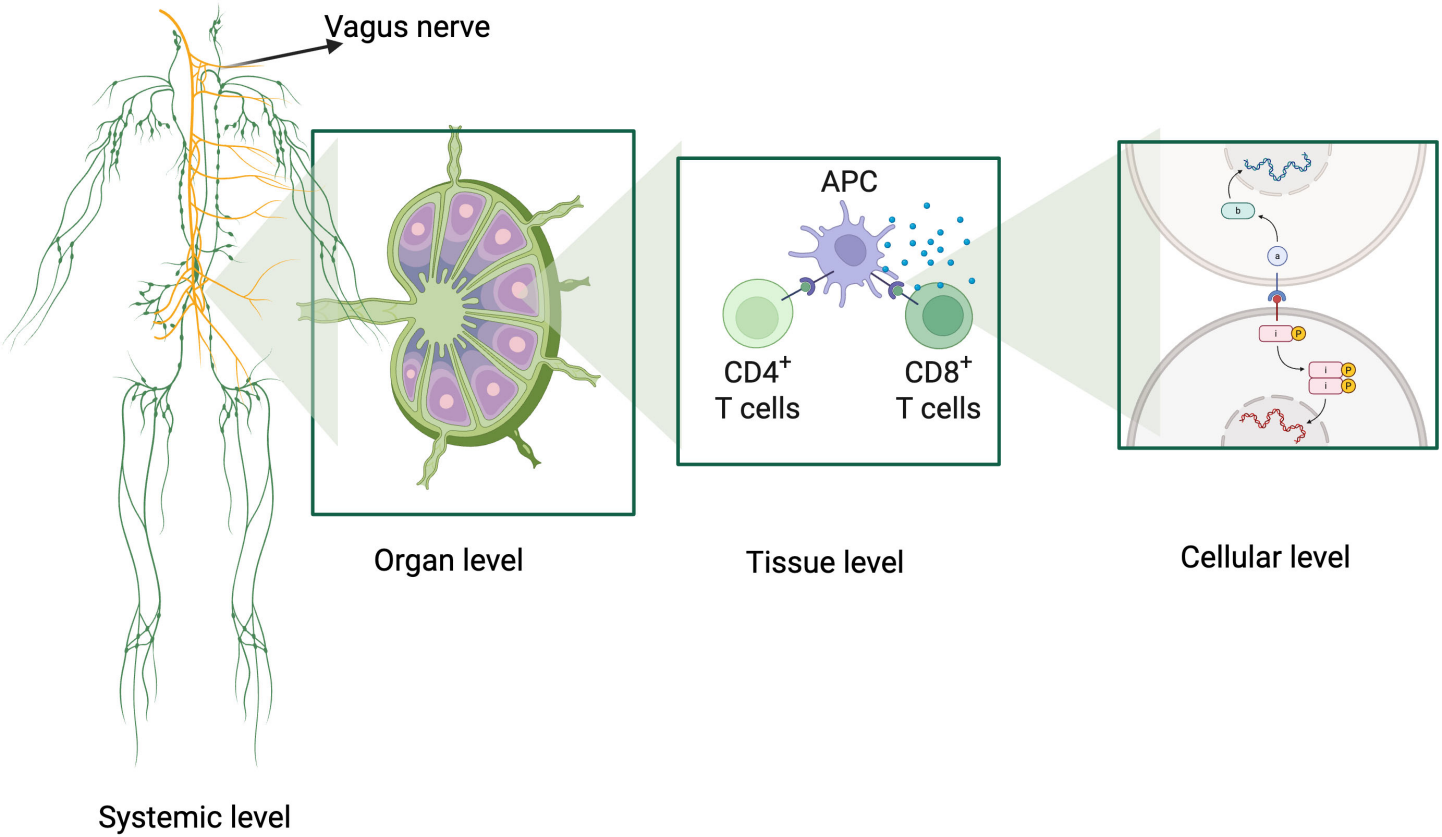
Graphical abstract illustrating the multiscale organization and information processing of the immune system. The figure highlights four interconnected levels: 1) Systemic Level: integration of the vagus nerve, showcasing the neuroimmune axis and its role in modulating immune responses; 2) Organ Level: representation of the lymph node structure as a hub for antigen processing, immune cell interaction, and coordination; 3) Tissue Level: detailed view of antigen-presenting cells (APCs) interacting with CD4+ and CD8+ T cells, emphasizing the role of cytokine signaling in cellular activation; 4) Cellular Level: intracellular processes including antigen recognition, signal transduction pathways (e.g., NF-κB, JAK-STAT), and gene expression. Created using BioRender, this figure encapsulates the dynamic, hierarchical organization of immune processes, linking systemic coordination to molecular specificity. Created in https://BioRender.com.

The immune system represents one of the most sophisticated biological networks in nature: a multiscale information processor that operates simultaneously at the molecular, cellular, tissue, and systemic levels to coordinate real-time adaptive responses (1). This network exhibits emergent properties that transcend the capacities of its individual components, generating a collective system capable of learning, remembering, and continuously evolving in the face of changing environmental challenges (2) (**Figure 1**).

### 1.2 Antifragility: beyond conventional robustness

Unlike merely robust systems that resist perturbations, the immune system exemplifies the concept of antifragility, as described by Nassim Nicholas Taleb (2): the capacity of a system to benefit from stressors, volatility, and disorder, emerging stronger and more capable after each challenge. This property is manifested in fundamental processes such as:

- Somatic hypermutation: where exposure to antigens triggers targeted mutations that improve antibody affinity.
- Clonal selection: which amplifies the most effective responses while eliminating suboptimal ones.
- Immunological memory: which transforms each pathogenic encounter into a permanent learning opportunity.
- Trained immunity: in which innate cells develop epigenetic memory that enhances future responses.
- Operation at the critical state: dynamic optimization

The immune system operates in a dynamic regime close to a critical state—a point of equilibrium between excessive order and chaotic disorder (3). This critical state maximizes:

- Sensitivity to relevant signals while filtering out environmental noise (4).
- Controlled amplification of minimal threats into effective and proportionate responses (5).
- Adaptive plasticity without compromising organismal stability (6).

This critical operation is underpinned by fundamental principles of information processing:

- Clonal diversity to maximize recognition potential (entropy) (7).
- Functional redundancy to ensure resilience through overlapping pathways (8).
- Non-local signaling networks that coordinate global responses from local triggers (9).
- Metabolic and epigenetic regulation as additional layers of control and memory (3)

#### 1.2.1 Unifying conceptual framework: canonical processing functions

To deconstruct this complexity, we propose a unifying framework based on two complementary conceptual layers:

Layer 1: Universal canonical functions

At every scale, the immune system executes six canonical information-processing functions (4, 5):

- Sensing: detection of molecular and cellular signals (6, 7)
- Coding: translation of signals into specific molecular patterns (10)
- Decoding: interpretation of patterns into functional programs (10)
- Response: execution of coordinated biological actions (11)
- Feedback: dynamic adjustment based on outcomes (12)
- Learning: adaptation of future responses based on experience (13)

Layer 2: Emergent organizational principles

These canonical functions are organized according to principles that emerge from complex network theory (8, 9):

- Criticality: Operation in dynamic regimes that optimize processing (14)
- Modularity: Organization into specialized functional subunits (15)
- Centrality: Critical nodes that integrate and coordinate information (16)
- Small-world topology: Efficient connections that facilitate global coordination (17)
- Redundancy: Multiple pathways that ensure fault tolerance (18)

Multiscale Integration: From Molecular to Systemic

This conceptual framework coherently unfolds across multiple biological scales:

- Molecular scale: Receptors, signaling pathways, and post-translational modifications (19)
- Cellular/tissue scale: Immunological synapses, germinal centers, and specialized microenvironments (20)
- Systemic/neuroimmune scale: Integration with the nervous, endocrine, and microbiota systems (21)

Each scale exhibits the same canonical functions but with specific implementations that reflect the constraints and opportunities of its particular organizational level.

- Toward a Unified Theory of Immunological Information Processing

This transdisciplinary approach integrates concepts from:

- Information theory and signal processing (22).
- Physics of complex systems and phase transitions (23).
- Network theory and topological analysis (24).
- Systems biology and precision medicine (25).

The goal is to develop a predictive understanding that not only explains immunological behavior but also enables the rational design of interventions that leverage the fundamental principles of biological information processing (26).

## 2 Multiscale processing of immunological information

The immune response is orchestrated through a complex integration of signals, processes, and feedback loops that operate at different levels of organization, from the molecular detection of antigens to systemic coordination (27). This section explores how each scale contributes to shaping a network of interactions critical for the antifragility of the immune system (**Table 1**).

**Table 1:** Specific examples of how the canonical functions of immune information processing are manifested at the molecular, cellular/tissue, and systemic/neuroimmune scales.

Canonical Function	Molecular Scale	Cellular/Tissue Scale	Systemic/Neuroimmune Scale
Sensing	PRRs (TLRs, NLRs), TCR/BCR recognizing PAMPs, DAMPs, specific antigens.	Dendritic cells and macrophages sensing antigens and microenvironmental cues.	Nervous system detecting inflammation via the vagus nerve; systemic detection of inflammatory signals (circulating cytokines).
Coding	Signaling cascades (JAK-STAT, NF-κB, MAPK); protein phosphorylation; second messengers (Ca^2^⁺, cAMP).	Immunological synapse; paracrine/autocrine cytokine and chemokine signaling; germinal center formation.	Coding of immune signals into neural patterns (neuroimmune networks); transmission via hormonal and metabolic signals.
Decoding	Activation of transcription factors (NF-κB, STATs, AP-1); nuclear translocation and epigenetic regulation.	Integrated cellular decisions: proliferation, differentiation, anergy, apoptosis; clonal selection in germinal centers.	Central neuroimmune integration: brain interpretation of peripheral immune signals and regulation of sickness behavior (fever, fatigue).
Response	Production and release of cytokines, chemokines, antibodies, effector molecules (granzymes, perforin).	Cell migration, cytotoxicity, phagocytosis, secretion of local antibodies and cytokines.	Coordinated physiological responses: fever, systemic inflammation, metabolic changes; activation of the hypothalamic-pituitary-adrenal (HPA) axis.
Feedback	Molecular inhibitors: SOCS, IκB, immune checkpoints (PD-1, CTLA-4). Positive feedback via proinflammatory cytokines (IL-2, IFN-γ).	Regulatory cells (Tregs, myeloid-derived suppressor cells); local gradients of regulatory (IL-10, TGF-β) and proinflammatory cytokines.	Neuroendocrine feedback via the HPA axis; central regulation by the vagus nerve and inflammatory reflex; systemic modulation by the gut microbiota.
Learning	Lasting epigenetic changes (methylation, acetylation); stable transcriptional reprogramming; somatic gene editing.	Formation of immunological memory: memory T/B cells, tissue-resident memory; functional plasticity of innate cells (trained immunity).	Sustained neuroimmune adaptation: conditioned learning of the immune system, persistent modulation of immune activity by prior experiences (stress, microbiota).

This table highlights the functional continuity and material specificity with which each canonical function operates across different biological levels, facilitating an integrated understanding of systems immunology from a multiscale perspective.

### 2.1 Molecular scale: foundations of immunological information processing

#### 2.1.1 Introduction: the molecular level as a computational basis

The immune system operates as a distributed computational network whose information processing capacity fundamentally emerges from the molecular scale. At this level, proteins, nucleic acids, and lipids function as basic processing elements, equivalent to the microprocessors of a biological computational system. Each immune cell contains thousands of these “molecular microprocessors” working in parallel, integrating complex environmental signals and translating this information into specific functional response**s** (28, 29).

The critical importance of this scale lies in the fact that it is here where the molecular specificity of immune recognition is defined and where the plastic properties that allow the system to adapt to new challenges emerge. Each receptor, enzyme, and transcription factor acts as a processing node that not only receives and transmits information but also qualitatively transforms it. This capacity for information transformation is what enables relatively simple signals (presence/absence of ligand) to be converted into complex activation patterns that determine the functional fate of each immune cell (30, 31).

#### 2.1.2 Architecture and typology of molecular receptors

##### 2.1.2.1 Sensing and recognition

The architecture of the immune molecular recognition system is organized into families of specialized receptors, each optimized to detect different types of biological information. Pattern recognition receptors (PRRs) constitute the first line of sensing, detecting pathogen-associated molecular patterns (PAMPs) and endogenous damage signals (DAMPs). This family includes Toll-like receptors (TLRs), RIG-I-like receptors (RLRs), NOD-like receptors (NLRs), and cytosolic DNA sensors, each specialized in recognizing different types of molecular threats.

T-cell receptors (TCRs) and B-cell receptors (BCRs) represent the adaptive recognition system, capable of generating virtually unlimited molecular diversity through gene recombination. These receptors not only recognize specific antigens but also integrate this information with contextual signals provided by costimulatory and inhibitory receptors. The CD28/CTLA-4 family, PD-1/PD-L1, and costimulatory molecules such as CD40, OX40, and 4-1BB form feedback circuits that modulate the intensity and duration of the immune response (30, 32).

The spatial topology of these receptors on the cell membrane is not random. They are organized into lipid microdomains (lipid rafts) that facilitate the functional interaction between related receptors. Receptor clustering allows for the amplification of weak signals and the integration of multiple simultaneous inputs. This spatial organization creates a computational architecture where physical proximity determines functional connectivity, similar to the architecture of integrated circuits in electronic systems (33).

##### 2.1.2.2 Signal integration and hierarchy

The immune cell must constantly integrate multiple simultaneous signals, each with different informational weight and contextual relevance. This integration process follows hierarchical principles where certain signals act as “master signals” that can override or modulate other inputs. For example, TCR signals provide the basic antigenic specificity, but their final interpretation critically depends on the simultaneous presence of costimulatory signals.

Costimulation functions as a verification system that confirms the legitimacy of activation. Without appropriate costimulatory signals, even strong antigenic signals can result in anergy or tolerance. Conversely, inhibitory signals act as molecular brakes that can halt responses even in the presence of strong activating stimuli. This multi-checkpoint architecture creates a robust system against inappropriate activations (34, 35).

Functional redundancy in the receptor system provides robustness against individual failures but also creates complexity in integration. Multiple receptors may detect the same type of threat, but each can activate slightly different signaling pathways, resulting in functional nuances that allow for more precise and context-adapted responses (36).

#### 2.1.3 Molecular encoding: biochemical translation of the signal

##### 2.1.3.1 Signaling cascades

The main immune signaling pathways—NF-κB, JAK-STAT, and MAPK—function as amplification and processing systems that translate molecular recognition events into functional cellular changes. Each pathway has a distinct computational architecture optimized for different types of information processing (37).

**Figure 2:**
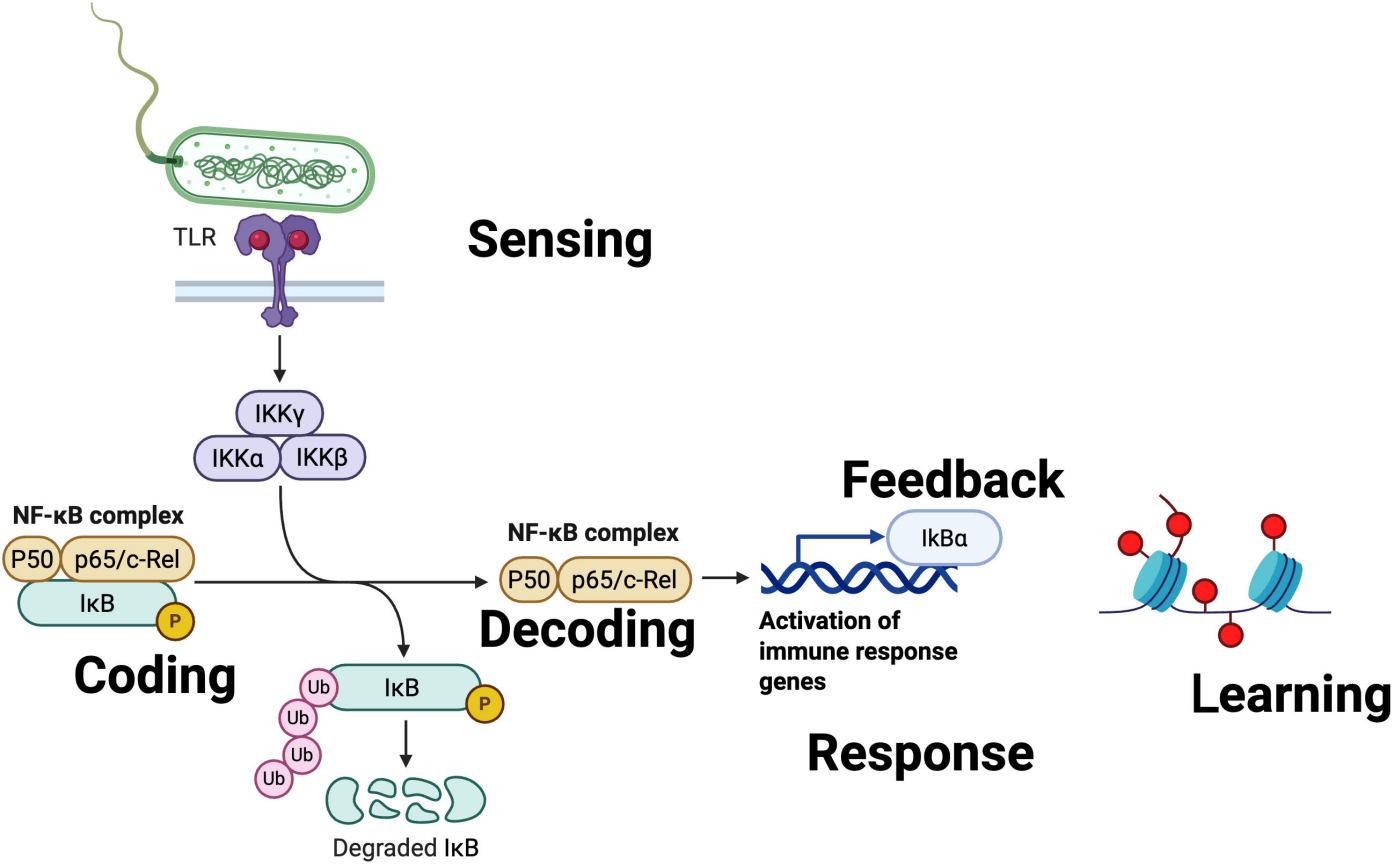
Immune signal processing at the molecular and subcellular scale: the canonical NF-κB pathway. This figure illustrates the logical and biochemical sequence of events that constitute information processing within an immune cell, exemplified by the canonical NF-κB pathway—one of the principal regulatory axes of innate and adaptive immunity. The process begins with the sensing of pathogen-associated molecular patterns (PAMPs) through pattern recognition receptors (PRRs), such as Toll-like receptors (TLRs) on the plasma membrane, which trigger signal transduction into the cytoplasmic compartment. The signal is then coded by the sequential activation of the IKK complex (IKKα, IKKβ, and IKKγ/NEMO), resulting in the phosphorylation and ubiquitination of IκB, targeting it for proteasomal degradation. Once IκB is degraded, the NF-κB complex is released (decoding) and translocates to the nucleus, where it decodes the signal by binding to specific DNA regulatory elements and activating target gene programs. The functional response is reflected in the expression of genes encoding cytokines, chemokines, and survival regulators, orchestrating the immune response. Feedback is achieved through the induction of IκBα, which, once synthesized, returns to the cytoplasm to sequester NF-κB and thus limit the duration and magnitude of the response via a negative feedback loop. Finally, learning is manifested in the induction of epigenetic modifications, such as histone and DNA changes, which establish a molecular memory capable of modulating gene accessibility and the efficiency of future immune responses. Created in https://BioRender.com.

The NF-κB pathway operates as a rapid response system for stress and inflammatory signals. Its architecture includes multiple amplification points: from the initial activation of the IKK complex to the nuclear release of NF-κB, each step can amplify the initial signal by several orders of magnitude. Critically, NF-κB exhibits oscillatory dynamics that encode temporal information—the frequency and amplitude of these oscillations determine which genes are activated and to what extent (38–40) (**Figure 2**).

JAK-STAT pathways provide a direct mechanism for translating cytokine signals into specific transcriptional changes. Different combinations of cytokine receptors activate different STATs, creating a “STAT code” that enables specific transcriptional responses for each signaling context. Specificity arises from which STATs are activated, their post-translational modifications, and cooperating transcription factors (41, 42).

MAPK pathways (ERK, JNK, p38) process information about cellular stress, growth, and differentiation signals. Their cascade architecture allows for both amplification and integration of multiple inputs. “Molecular codes” emerge from the specific combination of activated MAPKs, the duration of their activation, and the specific substrates they phosphorylate (42, 43).

##### 2.1.3.2 Post-translational modifications

Post-translational modifications function as a molecular language, allowing the same proteins to adopt multiple functional states depending on cellular context. Phosphorylation acts as the fastest and most reversible mechanism, enabling functional changes on time scales of seconds to minutes. Phosphorylation patterns create molecular “barcodes” that determine protein-protein interactions, subcellular localization, and enzymatic activity (44).

Ubiquitination provides both degradation signals and signals for localization and functional modification. Different types of ubiquitin chains (K48, K63, K11) encode different information: K48 typically signals proteasomal degradation, while K63 can activate signaling or specific localization. This diversity enables the same biochemical mechanism to encode multiple types of information (45).

Acetylation and methylation modifications, particularly on histones, create long-term molecular memory that persists beyond the initial signaling. These modifications establish chromatin states that influence gene accessibility during future responses, providing a molecular basis for immunological memory and innate immune training (46).

#### 2.1.4 Molecular decoding: interpretation and functional programs

##### 2.1.4.1 Transcription factors and signal reading

Transcription factors act as molecular decoders that interpret integrated biochemical signals and translate them into specific gene programs. NF-κB does not act alone, but in combination with other factors such as AP-1, IRFs, and STATs to create “combinatorial codes” that determine specific gene expression patterns (47).

Specificity emerges at various levels of organization: differential affinity for specific DNA sequences, cooperative interactions between transcription factors, and availability of binding sites determined by chromatin state. For example, the difference between pro-inflammatory activation and anti-inflammatory resolution may depend on whether NF-κB associates with co-activators like p300 or co-repressors like HDAC (48).

Combinations of signals create a molecular Boolean logic where different combinations of active transcription factors result in different cellular fates. The presence of FOXP3 plus TGF-β plus IL-2 promotes differentiation toward regulatory T cells, whereas IL-12 plus IFN-γ promotes Th1 differentiation. This combinatorial logic allows a limited number of transcription factors to generate a much greater diversity of functional responses (49).

##### 2.1.4.2 Epigenetic and chromatin regulation

Chromatin functions as a molecular memory system that retains information about past immunological experiences. Chromatin states—euchromatic (active) versus heterochromatic (repressed)—determine which genes are available for rapid activation versus those requiring extensive remodeling before expression (50).

Epigenetic marks create a histone code that is interpreted by specialized proteins. H3K4me3 marks active promoters, H3K27me3 marks genes that are repressed but poised for activation, and H3K9me3 marks constitutive heterochromatin (51). The combination of these marks on different genes creates an “epigenetic landscape” that influences future responses (52).

Importantly, some epigenetic marks persist after the initial signaling has ceased, providing a molecular basis for innate immune “training” and certain types of immunological memory. Cells that have experienced prior activation retain epigenetic marks that facilitate faster and more robust responses to subsequent stimuli, including related but non-identical stimuli (52).

#### 2.1.5 Spatiotemporal dynamics of molecular signaling

##### 2.1.5.1 Subcellular organization and microdomains

The spatial compartmentalization of molecular signaling is critical for the specificity and efficiency of information processing. Lipid rafts concentrate specific receptors and their associated signaling machinery, creating microdomains where specific signals can be processed without interference from other pathways (53). This organization is particularly important for receptors that require clustering for activation, such as TCRs and BCRs (54).

Signaling complexes (signalosomes) are dynamic structures assembled in response to specific stimuli. For example, the inflammasome is a multiprotein complex that assembles in response to danger signals, providing a platform for caspase-1 activation and the release of pro-inflammatory cytokines (55). The formation of these complexes allows for both signal amplification and specificity through the selective recruitment of specific components (56).

Subcellular compartmentalization also includes specialized organelles such as the autophagosome, which not only degrades cellular material but also serves as a signaling platform for pathways like mTOR and AMPK (57). This spatial organization enables cells to integrate information about their metabolic state with their immune activation status (58).

##### 2.1.5.2 Temporality and signal dynamics

The temporal dimension of molecular signaling is as important as its intensity. Oscillations in the activity of transcription factors such as NF-κB are not noise, but temporally encoded information. The frequency of these oscillations determines which subsets of genes are activated—genes with high-affinity promoters respond to brief pulses, while genes with low-affinity promoters require sustained activation (59).

Temporal codes also include the relative timing of different signals. The temporal sequence in which signals are received can be as important as their presence or absence. For example, danger signals preceding antigenic signals promote immunity, while antigenic signals preceding danger signals can promote tolerance (60).

Integration of simultaneous versus sequential signals requires different molecular machinery. Simultaneous signals may be integrated through direct protein-protein interactions or post-translational modifications that alter activity. Sequential signals require molecular memory systems, typically based on epigenetic modifications or changes in levels of key proteins that persist after the first signal has ceased (61).

#### 2.1.6 Feedback regulation: dynamic balance

##### 2.1.6.1 Positive feedback

Positive feedback circuits in the immune system create amplification mechanisms that can convert weak initial signals into robust responses. Autoregulation is common in many immune signaling pathways—for example, NF-κB activates the transcription of its own components, creating an amplification loop that sustains the inflammatory response (62).

Costimulation mechanisms also create positive feedback. T cell activation not only requires TCR signals but also induces the expression of additional costimulatory receptors and their ligands, amplifying the initial response (63). This process creates activation thresholds—once a certain threshold is surpassed, positive feedback ensures a robust response.

Cytokine cascades represent perhaps the most dramatic example of positive feedback in immunology. Cytokines released by activated cells can activate additional cells, which release more cytokines, creating amplification cascades (64). These cascades can be beneficial for antipathogen responses but can also result in pathology when dysregulated.

##### 2.1.6.2 Negative feedback

Negative feedback mechanisms are essential to prevent excessive immune responses and to resolve inflammation after the threat has been eliminated (65). SOCS proteins (Suppressors of Cytokine Signaling) are induced by cytokine signaling and subsequently inhibit the same signaling, creating an automatic negative feedback loop (66).

Inhibitory receptors such as PD-1, CTLA-4, and TIM-3 provide molecular brakes that can stop immune activation even in the presence of strong stimulatory signals. These receptors are particularly important in contexts where excessive immune activation could result in tissue damage or autoimmunity (67).

Phosphatases and other regulatory enzymes provide rapid mechanisms for terminating activation signals. For example, phosphatases such as SHP-1 and SHIP can quickly deactivate tyrosine kinase-mediated signaling pathways, providing precise temporal control over activation signal duration (11).

#### 2.1.7 Criticality, molecular hubs, and network vulnerability

##### 2.1.7.1 Critical dynamics

The immune system exhibits features of self-organized critical systems, where small changes can result in activation avalanches that follow power laws. These critical dynamics are manifested in the distribution of immune response sizes—most stimuli result in small responses, but occasionally very large responses occur that follow a heavy-tailed distribution (68).

This critical organization provides significant adaptive advantages. It allows for extreme sensitivity to weak stimuli when necessary (such as detecting pathogens at low concentrations), while maintaining robustness against minor fluctuations that do not represent real threats. Energy efficiency is also optimized—the system can remain in a low-energy surveillance state but respond rapidly when needed (69).

Immune activation avalanches are observed in both innate and adaptive responses. In the innate system, activation of one dendritic cell can result in the activation of many T cells, which in turn activate B cells and more T cells, creating activation cascades. These avalanches follow statistical patterns similar to those observed in physical critical systems (70).

##### 2.1.7.2 Molecular hubs

Certain molecular nodes act as hubs that connect multiple signaling pathways, making them critical for system function but also creating vulnerabilities. PKR (protein kinase R) is an example of a hub connecting viral RNA recognition with interferon responses, apoptosis, and translational control. Its central position makes it critical for antiviral responses but also a target for viral evasion (71).

mTOR functions as a metabolic hub integrating signals about nutrient availability, growth factors, and cellular energy status with immune activation programs. T cells require mTOR activation for effector differentiation, but mTOR must also be inactivated for memory generation. This functional duality requires precise temporal regulation (72).

p53 acts as a hub integrating multiple types of cellular stress and determining whether cells survive or die. In the immune context, p53 is critical for preventing malignant transformation of cells that have experienced DNA damage during processes such as somatic hypermutation in B cells (73).

The topological importance of these hubs means that their dysfunction can have disproportionately large effects on the system. However, this centrality also makes them evolutionary targets for both pathogens seeking to subvert immune responses and therapies aiming to modulate immune function (74).

#### 2.1.8 Clinical and molecular engineering implications

A deep understanding of molecular immune information processing has direct implications for the development of new therapies. Rational CAR-T design benefits from knowledge of how different intracellular signaling domains influence T cell function. Next-generation CARs incorporate multiple costimulatory domains based on principles of molecular signal integration (75).

Molecular biosensors can be designed to detect specific immune activation states by monitoring key molecular markers. For example, biosensors that detect specific cytokine patterns or post-translational modifications can provide early diagnostics of immune dysfunction (76).

The identification of criticality biomarkers can enable the prediction of when the immune system is approaching critical transitions, such as the progression from acute to chronic inflammation or the development of autoimmunity. These biomarkers could include specific gene expression patterns, epigenetic modifications, or signaling dynamics (77).

Therapeutic interventions can be designed to modulate specific molecular hubs or to alter the topology of signaling networks. For example, specific kinase inhibitors can be used to modulate key signaling pathways, while epigenetic modulators can alter the molecular memory of the immune system (78).

#### 2.1.9 Integrative perspective and transition to the cellular scale

The complexity of molecular immune information processing reveals how the system’s emergent properties arise from the integration of multiple molecular processes. Molecular logic—based on specific recognition, signal integration, controlled amplification, and regulatory feedback—provides the fundamental principles that operate at all scales of the immune system (79).

The concepts of criticality, molecular hubs, and spatiotemporal dynamics explored at the molecular scale provide the foundation for understanding how individual immune cells process information and make functional decisions. Epigenetic modifications create molecular memory that influences future responses, while the spatial organization of receptors and signaling complexes determines the specificity and efficiency of information processing (80).

This molecular architecture lays the groundwork for organization and information processing at higher scales. Individual immune cells can be seen as biological computers whose “software” is determined by the molecular circuits we have discussed. The diversity of immune cell types reflects different molecular “programs,” each optimized for different aspects of immunological information processing (81).

**Figure 3:**
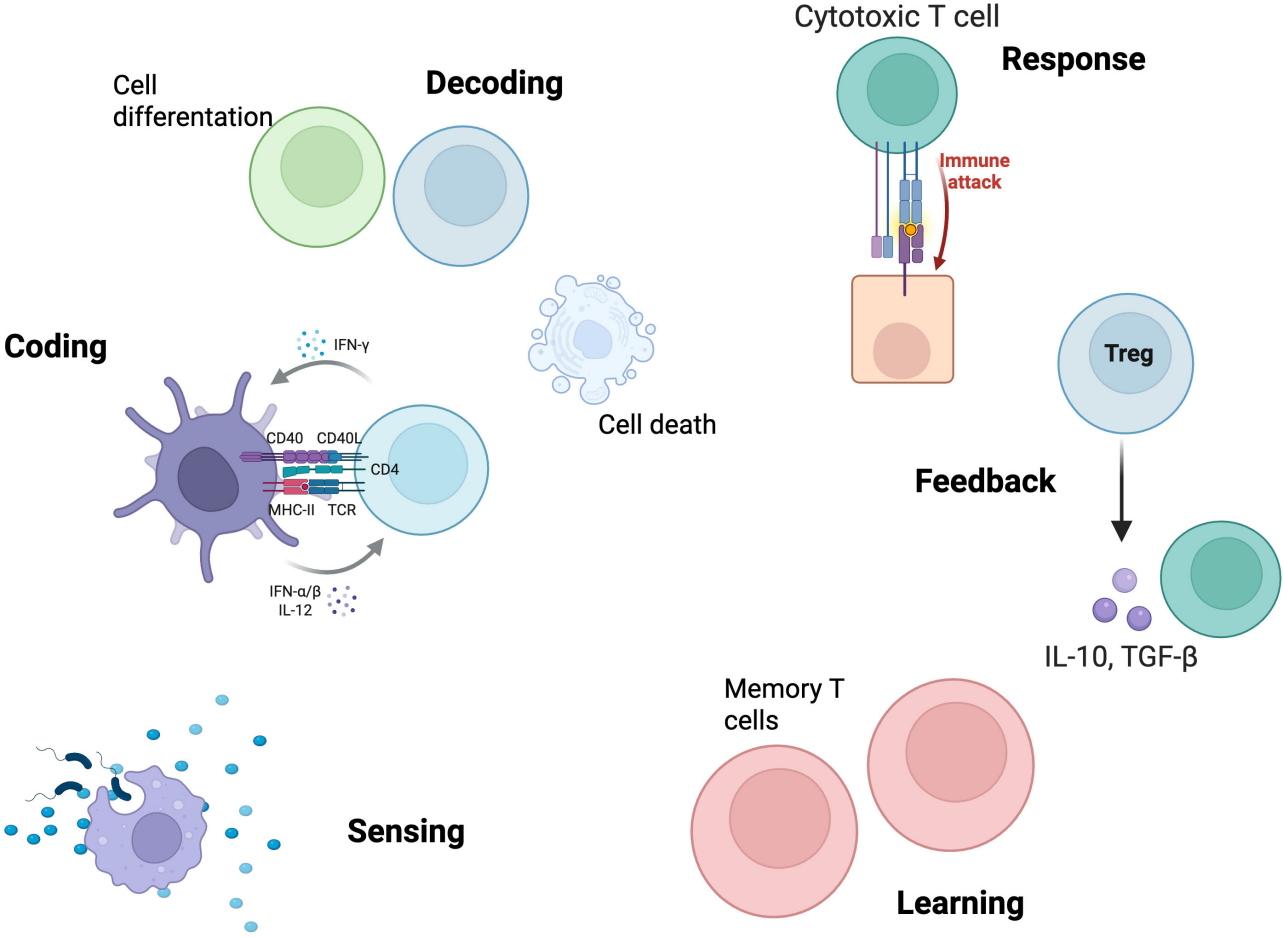
Canonical information processing functions at the cellular/tissue scale. This figure illustrates the sequence of canonical information processing functions executed by immune cells at the cellular/tissue level. The process begins with sensing, where antigen-presenting cells (APCs) detect and internalize antigenic material from the environment. Coding is mediated by the formation of the immunological synapse between APCs and T cells, integrating signals from major histocompatibility complex (MHC)-T cell receptor (TCR) interactions, co-stimulatory molecules (CD40/CD40L), and cytokines (IFN-α/β, IL-12, IFN-γ). Decoding encompasses cellular fate decisions, including cell proliferation, differentiation, and death, as a result of signal integration. The response is characterized by the activation of effector mechanisms, such as cytotoxic T cell-mediated immune attack. Feedback is provided by regulatory T cells (Treg), which secrete immunosuppressive cytokines (IL-10, TGF-β) to limit excessive immune activation and maintain tissue homeostasis. Learning is represented by the formation of memory T cells, ensuring rapid and robust responses upon subsequent antigen encounters. Together, these stages exemplify the emergent, context-dependent, and self-regulating nature of immune information processing at the cellular/tissue scale. Created in https://BioRender.com.

As we transition to the cellular and tissue scale, we will see how these molecular principles translate into complex cellular behaviors such as migration, differentiation, and intercellular communication. The spatial organization of cells in lymphoid tissues creates new levels of information processing that emerge from, but transcend, individual molecular logic. Cell-to-cell communication networks create distributed processing circuits where information is processed not only within individual cells but also across spatially organized cell populations (82) (**Figure 3**).

### 2.2 Cellular and tissue scale: collective intelligence and distributed coordination

At the cellular and tissue scale, the immune system undergoes a qualitative leap in its information processing capacity, advancing from the rigorous precision of molecular processing toward the emergence of collective intelligence and distributed coordination of remarkable complexity. This organizational level is not a mere aggregation of individual cells; rather, it constitutes a domain where cooperative behaviors, self-organizing patterns, and information circuits emerge that completely transcend the properties of isolated cells or purely molecular processes. Here, information processed at the molecular level is integrated, amplified, and transformed through densely interconnected cellular networks, configuring living architectures for adaptive decision-making (83).

The transition to the cellular-tissue scale entails a radical transformation in processing logic: emergent properties such as collective intelligence, distributed functional memory, adaptive plasticity, and the capacity for spatial and temporal self-organization appear. These capabilities enable the immune system to respond, adjust, and evolve far more effectively to complex and dynamic challenges, maintaining functional coherence and resilience even under conditions of extreme perturbation (84).

#### 2.2.1 Emergent transition: from molecular processing to collective intelligence

##### 2.2.1.1 Emergence of self-organization

The cellular-tissue scale marks a critical transition from the binary, linear, and local logic of molecular processing to the emergence of collective and organizational phenomena of great complexity. At this level, self-organization is a fundamental feature: immune cells do not merely respond to individual signals, but interact dynamically through cell-cell contacts, immunological synapses, and soluble signals, generating cooperative patterns and functional networks that self-assemble and reconfigure in real time (85).

Immunological synapses exemplify this leap in complexity: they not only act as nodes for the integration of molecular signals but also orchestrate the coordination of shared decisions, enabling cooperative amplification and emergent responses. Collective processing allows the robustness and adaptability of the system to vastly exceed the limitations of individual cells (86).

##### 2.2.1.2 Distributed network architectures

In this context, cellular interactions generate highly specialized network architectures that optimize the efficiency of distributed processing and information management. The small-world topology, characteristic of immune tissues, provides a platform where specialized local processing coexists with agile and efficient global coordination. Functional modules such as germinal centers, dendritic cell niches, or tissue microenvironments operate in parallel and communicate, integrating dynamically according to the demands of the immunological challenge (87).

This network architecture not only enhances response capacity but also facilitates the containment of local disturbances, efficient signal propagation, and synchronization of collective responses. The result is a system capable of self-organizing and scaling local responses to systemic dynamics, while preserving both functional diversity and the permanent capacity for adaptation (88).

#### 2.2.2 Distributed implementation of canonical functions

##### 2.2.2.1 SENSING: distributed and collaborative surveillance

The sensing function emerges as collaborative surveillance, in which specialized cellular populations—such as dendritic cells, macrophages, and B cells—establish interconnected early-detection networks capable of scanning large tissue volumes and distinguishing heterogeneous microenvironments. Through cell-cell contacts, soluble signals, and indirect intercellular communication (such as extracellular vesicles and nanotubes), efficient integration of environmental and antigenic information is achieved. Dendritic cell networks operate as sophisticated monitoring and alert systems, not only increasing sensitivity through functional overlap and distributive redundancy but also generating resilience to the loss or dysfunction of individual components. Thus, the immune system can detect, discriminate, and prioritize threats in real time, maintaining robust and adaptive surveillance in the face of environmental variability and the evolutionary pressure of emerging pathogens (89).

##### 2.2.2.2 ENCODING: cellular information maps

At this scale, encoding transcends mere molecular translation and is manifested in the active creation and modulation of chemokine and cytokine gradients. These gradients act as authentic dynamic maps of spatial and temporal information, defining migration routes, activation zones, and functional territories within tissues. Cellular communities not only perceive but also generate and readjust these maps, enabling contextual information to be precise, selective, and flexible. The interaction of multiple cells in the production and consumption of chemical signals enables redundant and polymodal encoding, increasing the capacity for representation and response to the complexity of immunological challenges. Thus, cellular encoding is not passive but rather a collective, self-organized process in continuous feedback with the microenvironment and the global state of the tissue (90).

##### 2.2.2.3 DECODING: consensus decisions

Tissue decoding is configured as an advanced consensus process in which multiple cellular populations integrate distributed information, both historical and contemporary, to generate robust, context-adapted collective responses. This mechanism is observed in germinal centers, where competition and cooperation between B cells, follicular T cells, and follicular dendritic cells lead to optimal decisions regarding affinity maturation, diversity generation, and memory establishment. It is not a simple summation of signals: cellular nodes execute nonlinear integration processes, filtering, weighting, and synthesizing information to maximize efficacy and minimize errors, such as autoreactivity or the generation of ineffective clones. Thus, tissue decoding is characterized by its consensus capacity, adaptive flexibility, and collective learning (91).

##### 2.2.2.4 RESPONSE: synchronization and collective coordination

The execution of immune responses at the tissue scale emerges as a phenomenon of distributed synchronization, in which different cell types (macrophages, lymphocytes, presenting cells, and stromal cells) self-assemble into functionally specialized structures. This assembly, exemplified by the formation of granulomas or germinal centers, optimizes the containment, neutralization, and resolution of immunological challenges. The collective response is regulated both spatially and temporally, adjusting the sequence and magnitude of effector actions via feedback mechanisms and contextual signals. Functional synchronization is essential to avoid dispersed or chaotic responses, promoting global tissue coherence and efficiency in the use of cellular and energetic resources (92).

##### 2.2.2.5 FEEDBACK: emergent homeostasis

Feedback mechanisms at the tissue level implement distributed and multilevel regulation, where multiple coupled circuits (positive and negative) interact dynamically. Regulatory cells such as Tregs, immune checkpoints, and contextual inhibitory signals work together to maintain system stability, preventing both harmful hyperactivation and excessive tolerance. Emergent homeostasis results from this dynamic equilibrium: the system continuously adjusts the intensity and duration of immune responses, modulating resilience, tolerance, and recovery after perturbations. Thus, self-regulation emerges not from central control, but from ongoing cooperation and local-global adaptation among cellular and molecular participants (93).

##### 2.2.2.6 LEARNING: distributed and adaptive memory

Immunological memory at the tissue scale is expressed as distributed collective intelligence, integrating prior experiences and functional states through dynamic networks of memory cells, naive cells, and effector cells. This incremental learning is sustained by reciprocal communication, epigenetic reprogramming, and functional reconfiguration induced by successive exposures to antigens or environmental stimuli. The adaptive memory network is in constant update, allowing the immune system to progressively refine its recognition, response, and containment strategies. The result is robust learning, capable of anticipating and adapting to changing challenges, supporting system resilience in the face of unexpected events or pathogen evolution (94).

#### 2.2.3 Emergent organizational principles

##### 2.2.3.1 Self-organized criticality

Operation near criticality states emerges spontaneously from local interactions, maximizing adaptability without centralized control. Germinal centers clearly represent this principle, continuously balancing exploration (somatic mutation) and exploitation (clonal selection) to maintain optimal functional diversity and specificity (95).

##### 2.2.3.2 Dynamic modularity

Tissue modularity emerges as cooperative specialization, where different regions of immune tissue perform dynamically integrated complementary functions. This organization enables efficient, flexible, and parallel processing, facilitating rapid adaptation to contextual changes (96).

##### 2.2.3.3 Emergent cellular hubs

Dendritic cells act as critical integrative hubs, mobilizing antigenic and contextual information to modulate systemic responses. Their centralized function maximizes efficiency in information transmission, ensuring rapid and coordinated systemic responses (97).

##### 2.2.3.4 Functional redundancy

Functional redundancy emerges through the diversity of different cellular subpopulations, ensuring robustness and fault tolerance. This redundancy not only protects against the loss of individual components but also increases the system’s global coverage through complementary and cooperative perspectives (98).

#### 2.2.4 Antifragility and distributed optimization

Immunological tissues display antifragility through collective processes and organizational circuits that transform perturbations, stress, or environmental challenges into opportunities for improvement and adaptive optimization. This phenomenon is especially evident in scenarios such as affinity maturation in germinal centers, where exposure to antigens and clonal selection induce somatic hypermutation, generating controlled diversification of antibody repertoires. The selective pressure exerted by new antigenic challenges not only increases antibody affinity and specificity but also fosters robust immunological memory and system functional plasticity. Likewise, trained immunity in innate immune cells demonstrates the system’s capacity to metabolically and epigenetically reprogram macrophages, NK cells, and others, enabling them to respond more efficiently to future encounters with pathogens. Each immunological challenge, far from weakening the system, stimulates reorganization and learning at the cellular and tissue scale, contributing to systemic strengthening and increasing the resilience and global adaptive capacity of the immune system. This logic of “improvement through perturbation” is a foundation of the concept of biological antifragility and explains the immune system’s remarkable ability to evolve and adapt over time, even in highly changing environments and facing emerging challenges (99).

#### 2.2.5 Spatiotemporal self-organization

Spatiotemporal self-organization in the immune context generates emergent functional patterns that are fundamental for the efficacy and specificity of the immune response. Paradigmatic examples of this phenomenon are granulomas, which form in response to chronic infections such as tuberculosis: these self-assembled multicellular structures concentrate and contain the pathogen, delineating functional compartments from chemical and mechanical signals that direct the positioning, activation, and differentiation of participating cell types. Similarly, the dynamic organization of germinal centers in secondary lymphoid organs exemplifies how interaction and directed migration of B cells, follicular T cells, and follicular dendritic cells generate specialized microenvironments where the selection and optimization of humoral responses occur. Temporal self-organization also manifests in the rhythms of activation and coordinated migration of lymphocytes, the establishment of cytokine gradients, and sequential waves of cellular differentiation, thus ensuring a logical and efficient sequence of immunological events. These self-organized patterns enable the immune system to adapt its architecture and function in real time to the immediate requirements of the pathological or physiological context, maximizing efficacy while minimizing energy expenditure and collateral damage (100).

#### 2.2.6 Multiscale integration

The cellular-tissue scale constitutes an essential and irreplaceable bridge between molecular precision and systemic coordination. At this level, locally generated information and signals can be amplified and propagated through circulatory routes, cytokine gradients, or migration of effector and regulatory cells, scaling local responses into systemic effects. In turn, systemic signals such as hormones, neurotransmitters, and endocrine mediators can modulate the tissue microenvironment, adjusting sensitivity, activation thresholds, and response magnitude in specific cellular niches according to the organism’s global needs. This multiscale integration ensures the overall functional coherence of the immune system and allows dynamic adaptability to challenges that may change across both time and space. Moreover, these processes of bidirectional adjustment and feedback between scales constitute the basis for long-term immunological homeostasis and resilience, enabling the system to respond proportionally and efficiently to stimuli of diverse nature and intensity, without losing the capacity to recover balance after each challenge (101).

### 2.3 Systemic and neuroimmune scale: multiscale integration, distributed computation, and emerging networks

#### 2.3.1 Introduction: from local coordination to distributed systemic computation

The transition from information processing at the cellular level to systemic coordination represents one of the most fascinating phenomena in systems biology. This emergent scalability transforms discrete molecular signals into coordinated responses involving multiple organs, physiological systems, and complex behaviors. The immune system does not operate as a collection of isolated cells but as a distributed computational network that processes information in a parallel, hierarchical, and adaptive manner (102).

The emergence of systemic properties arises from nonlinear interactions among local components, generating phenomena such as self-organized criticality, where small perturbations can propagate as cascades of controlled amplification. This architecture enables the organism to maintain homeostasis while preserving the capacity for rapid response to threats, simultaneously optimizing both sensitivity and specificity of immune recognition (9).

Distributed immune computation is characterized by its capacity for parallel processing, where different cellular populations process specific information while maintaining cross-communication through networks of cytokines, chemokines, neurotransmitters, and metabolites. This architecture confers emergent properties on the system, including collective memory, adaptive learning, and functional plasticity that transcend the individual capabilities of its components (103).

#### 2.3.2 Systemic implementation of canonical functions

##### 2.3.2.1 SENSING: integrated systemic surveillance

Systemic-scale sensing represents a conceptual revolution in our understanding of immune detection. Beyond classical molecular pattern recognition, the system develops contextual detection capabilities that integrate environmental, metabolic, neurological, and microbial signals. Immune sensors distributed across peripheral tissues act as a network of biological “antennas” that detect not only pathogens but also subtle changes in tissue homeostasis, oxidative stress, metabolic alterations, and disturbances in the microbial ecosystem (104).

Neuroimmune integration exponentially amplifies systemic sensing capacity. Immune cells express receptors for neurotransmitters, hormones, and neuropeptides, while neurons can detect cytokines and microbial products. This sensory convergence allows the system to distinguish between external and internal threats, modulating responses according to the physiological and emotional context of the organism (105).

##### 2.3.2.2 ENCODING: multimodal signal translation

Systemic encoding transcends simple signal transduction to establish a complex molecular language that integrates information from multiple sources. The hypothalamic-pituitary-adrenal (HPA) axis acts as a master translator, converting immune signals into neuroendocrine responses, while the inflammatory-vagal axis provides a rapid, bidirectional communication channel between the periphery and the central nervous system (106).

The gut microbiota emerges as a crucial encoder, translating environmental information (diet, xenobiotics, pathogens) into molecular signals that influence systemic immunity and brain function. Microbial metabolites act as signaling molecules that modulate gene expression in distant immune cells, establishing a form of long-range chemical communication that connects the external environment with internal homeostasis (107).

##### 2.3.2.3 DECODING: distributed contextual interpretation

Systemic decoding involves multiple processing centers that interpret encoded signals according to specific contexts. The central nervous system acts as a supreme integrator, combining immune information with sensory data, prior memories, and emotional states to generate appropriate responses. However, decoding is not exclusively centralized; immune-competent organs such as the spleen, lymph nodes, and Peyer’s patches locally process information and make autonomous “decisions” regarding the activation of specific response programs (108).

This architecture of distributed decoding enables adaptive responses that vary by tissue, threat type, and overall physiological state. The system’s interpretive plasticity allows the same molecular signal to elicit different responses depending on context, optimizing energy efficiency and minimizing collateral damage (109).

##### 2.3.2.3 RESPONSE: coordinated systemic orchestration

Systemic responses are complex biological symphonies in which multiple organs and systems act in concert to restore homeostasis. Fever, sickness behavior, nutrient redistribution, and sleep modulation are examples of integrated responses involving the nervous, endocrine, immune, and metabolic systems working synchronously (110).

The temporal coordination of these responses reveals sophisticated chronobiological precision, where circadian, ultradian, and infradian rhythms modulate immune activity according to evolutionarily optimized patterns. This timing maximizes response efficacy while minimizing metabolic costs and disruptive effects on essential physiological functions (111).

##### 2.3.2.4 FEEDBACK: systemic control circuits

Neuroimmune feedback mechanisms form the foundation of systemic self-regulation. The vagal inflammatory reflex represents a real-time control circuit that monitors peripheral immune activity and adjusts inflammatory responses via the release of acetylcholine and other anti-inflammatory neurotransmitters. This neuroimmune control system prevents uncontrolled escalation of inflammatory responses that could result in tissue damage or septic shock (112).

Metabolic feedback adds another dimension of control, where products of immune metabolism (lactate, ATP, adenosine) modulate the activity of neighboring and distant immune cells. This form of metabolic communication creates feedback networks that optimize energy resource usage and coordinate responses according to the availability of metabolic substrates (113).

##### 2.3.2.5 LEARNING: systemic memory and adaptive plasticity

Systemic learning transcends classical immune memory to include forms of plasticity involving multiple physiological systems. Neuroimmune memory allows previous immune experiences to permanently modify nervous system responses to immune stimuli, creating a form of “immune conditioning” that can persist for years (114).

Metabolic imprinting represents another form of systemic learning, where early exposures to pathogens, diets, or stress permanently remodel immune metabolism and the response to future challenges. This metabolic plasticity allows the system to adapt responses according to the individual’s exposure history, optimizing energy efficiency and response specificity (115).

#### 2.3.3 Emerging organizational principles in the systemic network

##### 2.3.3.1 Dynamic criticality: optimization of systemic sensitivity

The immune system operates in states of self-organized criticality that maximize its detection capacity while maintaining operational stability. This dynamic criticality allows abrupt transitions between resting and activated states, facilitating rapid responses to threats while preventing spontaneous activations that could result in autoimmunity or chronic inflammation (116).

Criticality is manifested in phenomena such as cytokine cascades, where the initial release of inflammatory mediators can be exponentially amplified or attenuated according to context-specific critical thresholds. This nonlinear architecture enables the system to scale responses proportionally to the magnitude and persistence of detected threats (117).

##### 2.3.3.2 Functional modularity: specialization and integration

The modular organization of the immune system combines functional specialization with systemic integration capacity. Each immune-competent organ acts as a specialized module with specific information processing capabilities while maintaining functional connectivity with other modules through molecular and neural communication networks (118).

This modularity allows for localized responses without compromising global systemic function, while also facilitating the propagation of critical information when coordinated responses are required. Modularity also confers robustness, allowing for functional compensation when individual modules are compromised by damage or disease (119).

##### 2.3.3.3 Hubs and integration nodes: centers of distributed control

Certain organs and anatomical structures emerge as high-centrality hubs in the systemic neuroimmune network. The brain—especially the hypothalamus and brainstem—acts as a supreme integration center that processes immune information and generates coordinated neuroendocrine responses. The vagus nerve represents a privileged communication channel linking the central nervous system to peripheral immune organs, facilitating reflex control of inflammatory responses.

The spleen emerges as a critical immunological hub, not only filtering blood-borne pathogens but also acting as an integration center that receives sympathetic nerve signals and generates systemic immune responses. The liver functions as a metabolic processor, integrating nutritional, immunological, and hormonal signals to modulate systemic metabolism and the acute phase of inflammatory responses (120).

##### 2.3.3.4 Small-world topology: efficient connectivity

The connectivity architecture of the neuroimmune system exhibits small-world network properties that optimize communication efficiency between distant components. This topology allows critical information to propagate rapidly across the network while maintaining local clustering that facilitates specialized processing within specific functional modules (121).

Small-world connectivity is evident in phenomena such as the rapid propagation of systemic cytokines during acute inflammatory responses, where locally released mediators can influence distant organs within minutes. This architecture also facilitates the synchronization of immune responses across multiple tissues, enabling precise temporal coordination of specialized functions.

##### 2.3.3.5 Redundancy and fault tolerance: systemic robustness

The neuroimmune system incorporates multiple levels of redundancy that confer fault tolerance and operational robustness. Parallel signaling pathways ensure that critical information can propagate even when individual channels are compromised. This redundancy is observed in the multiplicity of cytokines with overlapping functions, the existence of sympathetic and parasympathetic neural pathways with complementary effects, and the ability of different cell types to perform similar functions when necessary (122).

Fault tolerance also manifests in the system’s ability to functionally reorganize after injury or perturbation. This adaptive plasticity allows unaffected organs to compensate for loss of function in damaged tissues, maintaining essential immunological capacities even under adverse conditions.

#### 2.3.4 Antifragility and systemic plasticity

##### 2.3.4.1 Perturbation as an adaptive driver

The concept of antifragility finds its paradigmatic expression in the neuroimmune system, where controlled exposures to challenges strengthen and expand the system’s adaptive capabilities. Subclinical infections, moderate psychological stress, dietary variability, and diverse environmental exposures act as “training” stimuli that remodel the functional architecture of the systemic network, expanding its response repertoire and increasing operational efficiency (123).

This antifragility is evident in phenomena such as heterologous immunity, where exposure to specific pathogens can confer cross-protection against unrelated pathogens via activation of broad-spectrum innate immune programs. Diversity of exposures during critical developmental periods also programs immune reactivity patterns that persist throughout life, optimizing responses according to the microbial environment and specific selective pressures of the ecosystem (124).

##### 2.3.4.2 Network remodeling: structural and functional plasticity

Neuroimmune system plasticity includes both functional changes in existing components and structural remodeling of network connections. Immunological neuroplasticity enables immune experiences to permanently modify neural circuits that process immune information, creating somatic memories that influence future responses.

Metabolic plasticity represents another dimension of systemic adaptation, where cellular metabolism is remodeled according to patterns of energy demand and substrate availability. This metabolic reprogramming allows continuous optimization of energy efficiency and response capacity according to the organism’s specific environmental demands.

##### 2.3.4.3 Implications for health and disease

Understanding systemic antifragility has profound implications for our understanding of health and disease states. Immunological resilience emerges as a systemic property that depends not only on the competence of individual components but also on the integrity of communication networks and the capacity for adaptive reorganization.

Immunosenescence can be understood as a progressive loss of antifragility, where the system loses its adaptive capacity and becomes increasingly fragile in the face of perturbations. Neuroimmune diseases represent disruptions in inter-system communication, resulting in loss of coordination and the emergence of pathological feedback loops (125).

#### 2.3.5 Irreducible computation and multiscale modeling

##### 2.3.5.1 Complexity and the limits of reductionism

The behavior of the neuroimmune system exhibits properties of computational irreducibility that limit the predictive power of reductionist analysis. The emergence of complex systemic properties from nonlinear interactions among multiple components requires computational approaches capable of capturing complex network dynamics across multiple temporal and spatial scales.

This computational irreducibility is evident in phenomena such as individual variability in immune responses, where similar genotypes can generate dramatically different immune phenotypes depending on specific histories of environmental exposures. Personalizing therapeutic interventions therefore requires approaches capable of integrating genomic, epigenomic, metabolomic, and environmental information into sophisticated predictive models (126).

##### 2.3.5.2 Complex systems modeling: computational tools

The development of multiscale computational models represents a critical frontier in systems immunology. Agent-based models can capture emergent behaviors of cell populations, while network models can identify connectivity patterns that determine systemic properties. Integrating these approaches with machine learning techniques enables the identification of complex patterns in high-dimensional data that escape traditional analysis (127).

Immunological digital twins represent a promising application of these technologies, where personalized computational models can simulate individual immune responses to optimize therapeutic interventions. These models integrate multi-omic data with clinical and environmental information to generate patient-specific predictions of treatment responses, risk of adverse effects, and disease progression (128).

#### 2.3.6 Connection with the network of networks: evolutionary and transdisciplinary perspective

##### 2.3.6.1 Coevolution of adaptive systems

The current architecture of the neuroimmune system is the product of millions of years of coevolution with pathogens, microbial symbionts, and diverse environmental pressures. This evolutionary history has resulted in a complex adaptive system that incorporates multiple scales of information processing, from molecular recognition to complex collective behaviors (129).

Host-microbiota coevolution has been particularly influential in shaping the architecture of the neuroimmune system. Commensal microorganisms are not passive residents but active participants in communication networks that influence neurological development, immune function, and behavior. This evolutionary partnership has resulted in deep mutual dependencies that transcend simple host-pathogen relationships.

##### 2.3.6.2 Universal principles in complex systems

The organizational principles identified in the neuroimmune system have broader applicability to the design of complex adaptive systems. Self-organized criticality, functional modularity, small-world connectivity, and antifragility represent evolutionary solutions to fundamental problems of information processing, coordination, and adaptation that are relevant across multiple domains.

These principles can inform the design of intelligent networks in engineering, distributed control systems, artificial intelligence architectures, and economic models. The immune system’s ability to maintain robustness while preserving adaptability offers valuable insights for the development of artificial systems that must operate in complex and changing environments (130).

##### 2.3.6.3 Evolutionary bases of neuroimmune architecture

The multiscale architecture of the neuroimmune system reflects evolutionary solutions to fundamental survival challenges that have persisted for millions of years. Selective pressure from pathogens has driven the development of increasingly sophisticated recognition systems, while the need to conserve energy resources has favored the evolution of control and regulatory mechanisms that optimize the cost-benefit ratio of immune responses.

Host-pathogen coevolution has resulted in a molecular arms race that has continually expanded the repertoire of recognition and response strategies. This coevolutionary dynamic has not only shaped the diversity of the adaptive immune system but has also refined innate detection mechanisms to achieve extraordinary sensitivity and specificity. The evolutionary conservation of basic neuroimmune circuits across species suggests these represent optimal solutions to fundamental computational problems of biological information processing (131).

##### 2.3.6.4 Emergence of universal systemic properties

Comparative analysis of neuroimmune systems across the animal kingdom reveals universal organizational principles that transcend specific phylogenetic differences. Self-organized criticality emerges independently in systems as diverse as invertebrate immunity and mammalian neuromodulation, suggesting that this property represents a convergent solution for optimizing signal detection in noisy environments.

The functional modularity observed in the human neuroimmune system finds parallels in simpler organizations, from chemical detection circuits in bacteria to social alarm systems in eusocial insects. This evolutionary convergence toward modular architectures reflects fundamental advantages in terms of robustness, evolvability, and computational efficiency that are independent of system-specific complexity.

Small-world connectivity, initially described in human social networks, is consistently observed in biological cell communication networks, suggesting that this topology represents an optimal solution to the problem of balancing global efficiency with local specialization. The ubiquitous presence of this architecture in diverse biological systems indicates deep organizational principles that can inform the design of complex artificial systems (132).

## 3 Limitations and future perspectives

Despite significant advances in conceptualizing the immune system as a multiscale information processing network, substantive challenges remain that limit the full operationalization of this theoretical framework in experimental, clinical, and computational practice. These limitations open avenues for future research aimed at transforming immunology into a science grounded in universal physical principles, integrating the dynamics of symmetry, criticality, and hierarchical organization.

### 3.1 Conceptual and experimental limitations

One of the main challenges lies in the difficulty of precisely quantifying and modeling critical phenomena such as self-organized criticality, phase transitions, and symmetry breaking in human immune tissues. Current experimental tools, although rapidly evolving, still present limitations in real-time capture and adequate resolution of these phenomena in complex biological models, such as 3D organoids and “tissue-on-a-chip” systems (133).

Additionally, cellular heterogeneity, the stochastic nature of immune responses, and enormous inter-individual variability complicate data interpretation and the validation of theoretical predictions. The integration of multi-omic data (transcriptomics, epigenomics, metabolomics, proteomics) demands more robust algorithms and computational platforms capable of handling the dimensionality and complexity of the information generated.

### 3.2 Limitations in modeling and prediction

At the computational level, current models often excessively simplify real biological dynamics, which can introduce bias and limit predictive capacity—particularly in clinical settings where accuracy is critical. Agent-based models, multiscale complex networks, and machine learning strategies still face barriers regarding scalability, interpretability, and extrapolation of results.

The key challenge is to develop hybrid models capable of integrating empirical data from different scales and sources, incorporating both cellular heterogeneity and the evolutionary and environmental history of each individual. This requires transdisciplinary collaborations and the design of “multilevel” experiments where in silico, *in vitro*, and *in vivo* approaches interact in cycles of iterative validation (134).

### 3.3 Translational and clinical limitations

The lack of standardized metrics for measuring phenomena such as criticality or symmetry dynamics in clinical data restricts the applicability of these concepts in medical practice. Robust, reproducible, and easily accessible biomarkers are needed to capture the dynamic state of the immune system in real patients.

Clinical translation of these approaches also requires overcoming fragmentation among disciplines—biology, physics, engineering, and computational sciences—by promoting integrated, interoperable, and open research platforms.

### 3.4 Future perspectives

To advance toward an immunology grounded in physical and information theory principles, we propose several strategic directions:

- Advanced Technological Development: Implement technologies for the capture of critical events and symmetry dynamics in complex biological systems, prioritizing models such as immunological organoids, artificial tissues, and microfluidic platforms with real-time sensors.
- New Theoretical and Computational Frameworks: Develop hybrid models that integrate network dynamics, machine learning, and information theory to describe transitions across scales and the emergence of collective properties. The concept of a “digital twin immunology” represents a promising frontier for the personalized simulation of immune responses and the prediction of clinical outcomes.
- Multiscale and Iterative Validation: Design coordinated studies that combine in silico, *in vitro*, and *in vivo* data to refine and falsify hypotheses regarding symmetry, criticality, and antifragility. This approach is key to transcending merely descriptive validation and advancing toward a predictive and manipulable immunological science.
- Development of Functional Metrics and Biomarkers: Prioritize the design of quantitative biomarkers for criticality, symmetry, and redundancy, applicable to multi-omic and phenotypic data, which can be used in clinical practice for risk stratification, prognosis, and personalized therapy.
- Transdisciplinarity and Open Platforms: Foster collaborative networks integrating immunologists, physicists, mathematicians, engineers, clinicians, and data scientists within open research platforms capable of accelerating cross-validation of concepts and technology transfer to the healthcare sector.

## 4 Conclusions

The immune system emerges as a multiscale adaptive network capable of processing information through architectures that integrate molecular precision, collective cellular intelligence, and systemic coordination. This research has articulated a transdisciplinary conceptual framework that transcends the traditional boundaries of descriptive immunology, proposing that the canonical functions of information processing—sensing, encoding, decoding, response, feedback, and learning—constitute functional invariants that are preserved across all levels of biological organization, from molecules to neuroimmune networks.

At the molecular scale, the immune system employs signaling circuits and epigenetic mechanisms that function as biological microprocessors, generating the diversity, specificity, and plasticity underpinning recognition and adaptation. At the cellular and tissue scale, a distributed collective intelligence emerges that transforms self-organization, criticality, and modularity into capacities for optimization and antifragility, enabling the system to evolve and strengthen in the face of challenges.

At the systemic and neuroimmune level, the dynamic integration between the immune system, the nervous system, and the microbiota enhances global plasticity, memory, and resilience. This multiscale perspective not only explains how precise responses are coordinated to complex threats, but also paves the way for designing advanced therapeutic strategies grounded in universal principles: symmetry and its breaking, self-organized criticality, functional modularity, small-world topology, and robust redundancy.

The proposed approach demonstrates that immunological complexity can be understood, modeled, and eventually intervened upon through a unified vision inspired by the physics of complex systems, information theory, and network biology. This perspective not only enriches basic research but also lays the foundation for the development of predictive computational models, functional biomarkers, and personalized clinical intervention strategies—crucial steps toward a truly integrative precision immunology.

## References

[1] Murphy FH. Review of antifragile: things that gain from disorder. Interfaces. (2014) 44:249–51. Available online at: https://www.jstor.org/stable/43699405 (Accessed January 15, 2025).

[2] Olivieri F Prattichizzo F Lattanzio F Bonfigli AR Spazzafumo L. Antifragility and antiinflammaging: Can they play a role for a healthy longevity? Ageing Res Rev. (2023) 84:101836. doi: 10.1016/j.arr.2022.101836, PMID: 36574863

[3] Chen X. From immune equilibrium to immunodynamics. Front Microbiol. (2022) 13:1018817. doi: 10.3389/fmicb.2022.1018817, PMID: 36504800 PMC9732466

[4] Sawicki J Berner R Loos SAM Anvari M Bader R Barfuss W. Perspectives on adaptive dynamical systems. Chaos Interdiscip J Nonlinear Sci. (2023) 33:071501. doi: 10.1063/5.0147231, PMID: 37486668

[5] Xu Z Song J Zhang H Wei Z Wei D Yang G. A mathematical model simulating the adaptive immune response in various vaccines and vaccination strategies. Sci Rep. (2024) 14:23995. doi: 10.1038/s41598-024-74221-x, PMID: 39402093 PMC11473516

[6] Medzhitov R Iwasaki A. Exploring new perspectives in immunology. Cell. (2024) 187:2079–94. doi: 10.1016/j.cell.2024.03.038, PMID: 38670066

[7] Chiffelle J Genolet R Perez MA Coukos G Zoete V Harari A. T-cell repertoire analysis and metrics of diversity and clonality. Curr Opin Biotechnol. (2020) 65:284–95. doi: 10.1016/j.copbio.2020.07.010, PMID: 32889231

[8] Moya A Ferrer M. Functional redundancy-induced stability of gut microbiota subjected to disturbance. Trends Microbiol. (2016) 24:402–13. doi: 10.1016/j.tim.2016.02.002, PMID: 26996765

[9] Su J Song Y Zhu Z Huang X Fan J Qiao J. Cell–cell communication: new insights and clinical implications. Signal Transduct Target Ther. (2024) 9:196. doi: 10.1038/s41392-024-01888-z, PMID: 39107318 PMC11382761

[10] Jia X He X Huang C Li J Dong Z Liu K. Protein translation: biological processes and therapeutic strategies for human diseases. Signal Transduct Target Ther. (2024) 9:44. doi: 10.1038/s41392-024-01749-9, PMID: 38388452 PMC10884018

[11] Quiroz RCN Philot EA General IJ Perahia D Scott AL. Effect of phosphorylation on the structural dynamics, thermal stability of human dopamine transporter: A simulation study using normal modes, molecular dynamics and Markov State Model. J Mol Graph Model. (2023) 118:108359. doi: 10.1016/j.jmgm.2022.108359, PMID: 36279761

[12] Rahman A Tiwari A Narula J Hickling T. Importance of feedback and feedforward loops to adaptive immune response modeling. CPT Pharmacomet Syst Pharmacol. (2018) 7:621–8. doi: 10.1002/psp4.12352, PMID: 30198637 PMC6202469

[13] Kato T Kobayashi TJ. Understanding adaptive immune system as reinforcement learning. Phys Rev Res. (2021) 3:13222. doi: 10.1103/PhysRevResearch.3.013222

[14] Cicchese JM Evans S Hult C Joslyn LR Wessler T Millar JA. Dynamic balance of pro- and anti-inflammatory signals controls disease and limits pathology. Immunol Rev. (2018) 285:147–67. doi: 10.1111/imr.12671, PMID: 30129209 PMC6292442

[15] Huitzil S Huepe C. Life’s building blocks: the modular path to multiscale complexity. Front Syst Biol. (2024) 4:1417800. doi: 10.3389/fsysb.2024.1417800 PMC12342035 40809143

[16] Tang K Tang J Zeng J Shen W Zou M Zhang C. A network view of human immune system and virus-human interaction. Front Immunol. (2022) 13:997851. doi: 10.3389/fimmu.2022.997851, PMID: 36389817 PMC9643829

[17] Liu B Xu M Gao L. Enhanced swarm intelligence optimization: Inspired by cellular coordination in immune systems. Knowl-Based Syst. (2024) 290:111557. doi: 10.1016/j.knosys.2024.111557

[18] Li N Kumar SRP Cao D Munoz-Melero M Arisa S Brian BA. Redundancy in innate immune pathways that promote CD8+ T-cell responses in AAV1 muscle gene transfer. Viruses. (2024) 16:1507. doi: 10.3390/v16101507, PMID: 39459842 PMC11512359

[19] Müller J Bayer FP Wilhelm M Schuh MG Kuster B The M. PTMNavigator: interactive visualization of differentially regulated post-translational modifications in cellular signaling pathways. Nat Commun. (2025) 16:510. doi: 10.1038/s41467-024-55533-y, PMID: 39779715 PMC11711753

[20] Yu X Pei W Li B Sun S Li W Wu Q. Immunosenescence, physical exercise, and their implications in tumor immunity and immunotherapy. Int J Biol Sci. (2025) 21:910–39. doi: 10.7150/ijbs.100948, PMID: 39897036 PMC11781184

[21] Lu S Zhao Q Guan Y Sun Z Li W Guo S. The communication mechanism of the gut-brain axis and its effect on central nervous system diseases: A systematic review. BioMed Pharmacother. (2024) 178:117207. doi: 10.1016/j.biopha.2024.117207, PMID: 39067168

[22] Haimovich A. Information theoretic signal processing and its applications [Bookshelf. IEEE Control Syst Mag. (2023) 43:97–109. doi: 10.1109/MCS.2023.3234387

[23] Tao P Du C Xiao Y Zeng C. Data-driven detection of critical points of phase transitions in complex systems. Commun Phys. (2023) 6:311. doi: 10.1038/s42005-023-01429-0

[24] Chen W-K. Topological analysis for active networks. IEEE Trans Circuit Theory. (1965) 12:85–91. doi: 10.1109/TCT.1965.1082396

[25] Chen R Snyder M. Systems biology: personalized medicine for the future? Curr Opin Pharmacol. (2012) 12:623–8. doi: 10.1016/j.coph.2012.07.011, PMID: 22858243 PMC4076046

[26] Vodovotz Y Xia A Read EL Bassaganya-Riera J Hafler DA Sontag E. Solving immunology? Trends Immunol. (2017) 38:116–27. doi: 10.1016/j.it.2016.11.006, PMID: 27986392 PMC5695553

[27] Grossman Z Meyerhans A Bocharov G. An integrative systems biology view of host-pathogen interactions: The regulation of immunity and homeostasis is concomitant, flexible, and smart. Front Immunol. (2023) 13:1061290. doi: 10.3389/fimmu.2022.1061290, PMID: 36761169 PMC9904014

[28] Pappalardo F Russo G Reche PA. Toward computational modelling on immune system function. BMC Bioinf. (2020) 21:546, s12859–020–03897–5. doi: 10.1186/s12859-020-03897-5, PMID: 33308137 PMC7733695

[29] Rapin N Lund O Bernaschi M Castiglione F. Computational immunology meets bioinformatics: the use of prediction tools for molecular binding in the simulation of the immune system. PloS One. (2010) 5:e9862. doi: 10.1371/journal.pone.0009862, PMID: 20419125 PMC2855701

[30] Mogensen TH. Pathogen recognition and inflammatory signaling in innate immune defenses. Clin Microbiol Rev. (2009) 22:240–73. doi: 10.1128/CMR.00046-08, PMID: 19366914 PMC2668232

[31] Patalano SD Fuxman Bass P Fuxman Bass JI. Transcription factors in the development and treatment of immune disorders. Transcription. (2025) 16:118–40. doi: 10.1080/21541264.2023.2294623, PMID: 38100543 PMC11970766

[32] Ong GH Lian BSX Kawasaki T Kawai T. Exploration of pattern recognition receptor agonists as candidate adjuvants. Front Cell Infect Microbiol. (2021) 11:745016. doi: 10.3389/fcimb.2021.745016, PMID: 34692565 PMC8526852

[33] Wang X Zhang Y Zhang R Zhang J. The diversity of pattern recognition receptors (PRRs) involved with insect defense against pathogens. Curr Opin Insect Sci. (2019) 33:105–10. doi: 10.1016/j.cois.2019.05.004, PMID: 31358188

[34] Cao FJ Feito M. Thermodynamics of feedback controlled systems. Phys Rev E. (2009) 79:41118. doi: 10.1103/PhysRevE.79.041118, PMID: 19518184

[35] Kang C-G. Origin of Stability Analysis: ”On Governors” by J.C. Maxwell [Historical Perspectives] (2016). Available online at: https://ieeexplore.ieee.org/document/7569049 (Accessed June 16, 2025).

[36] Minorsky. N. Directional stability of automatically steered bodies. J Am Soc Nav Eng. (1922) 34:280–309. doi: 10.1111/j.1559-3584.1922.tb04958.x

[37] Schmitz ML Weber A Roxlau T Gaestel M Kracht M. Signal integration, crosstalk mechanisms and networks in the function of inflammatory cytokines. Biochim Biophys Acta BBA Mol Cell Res. (2011) 1813:2165–75. doi: 10.1016/j.bbamcr.2011.06.019, PMID: 21787809

[38] Lawrence T. The nuclear factor NF- B pathway in inflammation. Cold Spring Harb Perspect Biol. (2009) 1:a001651–a001651. doi: 10.1101/cshperspect.a001651, PMID: 20457564 PMC2882124

[39] Li D Wu M. Pattern recognition receptors in health and diseases. Signal Transduct Target Ther. (2021) 6:291. doi: 10.1038/s41392-021-00687-0, PMID: 34344870 PMC8333067

[40] Hayden MS Ghosh S. NF-κB, the first quarter-century: remarkable progress and outstanding questions. Genes Dev. (2012) 26:203–34. doi: 10.1101/gad.183434.111, PMID: 22302935 PMC3278889

[41] Lv Y Qi J Babon JJ Cao L Fan G Lang J. The JAK-STAT pathway: from structural biology to cytokine engineering. Signal Transduct Target Ther. (2024) 9:221. doi: 10.1038/s41392-024-01934-w, PMID: 39169031 PMC11339341

[42] Hu Q Bian Q Rong D Wang L Song J Huang H-S. JAK/STAT pathway: Extracellular signals, diseases, immunity, and therapeutic regimens. Front Bioeng Biotechnol. (2023) 11:1110765. doi: 10.3389/fbioe.2023.1110765, PMID: 36911202 PMC9995824

[43] Nair B Menon A Rithwik Kalidas M Nath LR Calina D Sharifi-Rad J. Modulating the JAK/STAT pathway with natural products: potential and challenges in cancer therapy. Discov Oncol. (2025) 16:595. doi: 10.1007/s12672-025-02369-7, PMID: 40268770 PMC12018655

[44] Suskiewicz MJ. The logic of protein post-translational modifications (PTMs): Chemistry, mechanisms and evolution of protein regulation through covalent attachments. BioEssays. (2024) 46:2300178. doi: 10.1002/bies.202300178, PMID: 38247183

[45] French ME Koehler CF Hunter T. Emerging functions of branched ubiquitin chains. Cell Discov. (2021) 7:6. doi: 10.1038/s41421-020-00237-y, PMID: 33495455 PMC7835216

[46] Chen Y Liang R Li Y Jiang L Ma D Luo Q. Chromatin accessibility: biological functions, molecular mechanisms and therapeutic application. Signal Transduct Target Ther. (2024) 9:340. doi: 10.1038/s41392-024-02030-9, PMID: 39627201 PMC11615378

[47] Oh H Grinberg-Bleyer Y Liao W Maloney D Wang P Wu Z. An NF-κB transcription-factor-dependent lineage-specific transcriptional program promotes regulatory T cell identity and function. Immunity. (2017) 47:450–465.e5. doi: 10.1016/j.immuni.2017.08.010, PMID: 28889947 PMC5679261

[48] Karpinska MA Zhu Y Fakhraei Ghazvini Z Ramasamy S Barbieri M Cao TBN. CTCF depletion decouples enhancer-mediated gene activation from chromatin hub formation. Nat Struct Mol Biol. (2025), 1–14. doi: 10.1038/s41594-025-01555-z, PMID: 40360814 PMC12263429

[49] Freudenberg K Lindner N Dohnke S Garbe AI Schallenberg S Kretschmer K. Critical role of TGF-β and IL-2 receptor signaling in Foxp3 induction by an inhibitor of DNA methylation. Front Immunol. (2018) 9:125. doi: 10.3389/fimmu.2018.00125, PMID: 29456534 PMC5801288

[50] Mansisidor AR Risca VI. Chromatin accessibility: methods, mechanisms, and biological insights. Nucleus. (2022) 13:236–76. doi: 10.1080/19491034.2022.2143106, PMID: 36404679 PMC9683059

[51] Zhang T Cooper S Brockdorff N. The interplay of histone modifications – writers that read. EMBO Rep. (2015) 16:1467–81. doi: 10.15252/embr.201540945, PMID: 26474904 PMC4641500

[52] Al-Radhawi MA Tripathi S Zhang Y Sontag ED Levine H. Epigenetic factor competition reshapes the EMT landscape. Proc Natl Acad Sci. (2022) 119:e2210844119. doi: 10.1073/pnas.2210844119, PMID: 36215492 PMC9586264

[53] Mollinedo F Gajate C. Lipid rafts as signaling hubs in cancer cell survival/death and invasion: implications in tumor progression and therapy: Thematic Review Series: Biology of Lipid Rafts. J Lipid Res. (2020) 61:611–35. doi: 10.1194/jlr.TR119000439, PMID: 33715811 PMC7193951

[54] Rappazzo CG Fernández-Quintero ML Mayer A Wu NC Greiff V Guthmiller JJ. Defining and studying B cell and T cell receptor interactions. J Immunol Baltim Md 1950. (2023) 211:311–22. doi: 10.4049/jimmunol.2300136, PMID: 37459189 PMC10495106

[55] Xu Z Kombe Kombe AJ Deng S Zhang H Wu S Ruan J. NLRP inflammasomes in health and disease. Mol BioMed. (2024) 5:14. doi: 10.1186/s43556-024-00179-x, PMID: 38644450 PMC11033252

[56] Zhan X Li Q Xu G Xiao X Bai Z. The mechanism of NLRP3 inflammasome activation and its pharmacological inhibitors. Front Immunol. (2023) 13:1109938. doi: 10.3389/fimmu.2022.1109938, PMID: 36741414 PMC9889537

[57] Jeon S Jeon Y Lim J-Y Kim Y Cha B Kim W. Emerging regulatory mechanisms and functions of biomolecular condensates: implications for therapeutic targets. Signal Transduct Target Ther. (2025) 10:4. doi: 10.1038/s41392-024-02070-1, PMID: 39757214 PMC11701242

[58] Fleming A Bourdenx M Fujimaki M Karabiyik C Krause GJ Lopez A. The different autophagy degradation pathways and neurodegeneration. Neuron. (2022) 110:935–66. doi: 10.1016/j.neuron.2022.01.017, PMID: 35134347 PMC8930707

[59] Venkatachalam V Jambhekar A Lahav G. Reading oscillatory instructions: How cells achieve time-dependent responses to oscillating transcription factors. Curr Opin Cell Biol. (2022) 77:102099. doi: 10.1016/j.ceb.2022.102099, PMID: 35690043 PMC9533215

[60] Giles JR Globig A-M Kaech SM Wherry EJ. CD8+ T cells in the cancer immunity cycle. Immunity. (2023) 56:2231–53. doi: 10.1016/j.immuni.2023.09.005, PMID: 37820583 PMC11237652

[61] Satam H Joshi K Mangrolia U Waghoo S Zaidi G Rawool S. Next-generation sequencing technology: current trends and advancements. Biology. (2023) 12:997. doi: 10.3390/biology12070997, PMID: 37508427 PMC10376292

[62] Del Vecchio D Dy AJ Qian Y. Control theory meets synthetic biology. J R Soc Interface. (2016) 13:20160380. doi: 10.1098/rsif.2016.0380, PMID: 27440256 PMC4971224

[63] Chen L Flies DB. Molecular mechanisms of T cell co-stimulation and co-inhibition. Nat Rev Immunol. (2013) 13:227–42. doi: 10.1038/nri3405, PMID: 23470321 PMC3786574

[64] Jarczak D Nierhaus A. Cytokine storm—Definition, causes, and implications. Int J Mol Sci. (2022) 23:11740. doi: 10.3390/ijms231911740, PMID: 36233040 PMC9570384

[65] Ricklin D Lambris JD. Complement in immune and inflammatory disorders: pathophysiological mechanisms. J Immunol Baltim Md 1950. (2013) 190:3831–8. doi: 10.4049/jimmunol.1203487, PMID: 23564577 PMC3623009

[66] Baetz A Frey M Heeg K Dalpke AH. Suppressor of cytokine signaling (SOCS) proteins indirectly regulate toll-like receptor signaling in innate immune cells. J Biol Chem. (2004) 279:54708–15. doi: 10.1074/jbc.M410992200, PMID: 15491991

[67] Chen R-Y Zhu Y Shen Y-Y Xu Q-Y Tang H-Y Cui N-X. The role of PD-1 signaling in health and immune-related diseases. Front Immunol. (2023) 14:1163633. doi: 10.3389/fimmu.2023.1163633, PMID: 37261359 PMC10228652

[68] Hoffmann H Payton DW. Optimization by self-organized criticality. Sci Rep. (2018) 8:2358. doi: 10.1038/s41598-018-20275-7, PMID: 29402956 PMC5799203

[69] Munteanu C Schwartz B. The relationship between nutrition and the immune system. Front Nutr. (2022) 9:1082500. doi: 10.3389/fnut.2022.1082500, PMID: 36570149 PMC9772031

[70] Summerfield A McCullough KC. Dendritic cells in innate and adaptive immune responses against influenza virus. Viruses. (2009) 1:1022–34. doi: 10.3390/v1031022, PMID: 21994580 PMC3185519

[71] Schulz O Pichlmair A Rehwinkel J Rogers NC Scheuner D Kato H. Protein kinase R contributes to IFN-α/β production during viral infection by regulating IFN mRNA integrity. Cell Host Microbe. (2010) 7:354–61. doi: 10.1016/j.chom.2010.04.007, PMID: 20478537 PMC2919169

[72] Waickman AT Powell JD. mTOR, metabolism, and the regulation of T-cell differentiation and function. Immunol Rev. (2012) 249:43–58. doi: 10.1111/j.1600-065X.2012.01152.x, PMID: 22889214 PMC3419491

[73] Borrero LJH El-Deiry WS. Tumor suppressor p53: biology, signaling pathways, and therapeutic targeting. Biochim Biophys Acta Rev Cancer. (2021) 1876:188556. doi: 10.1016/j.bbcan.2021.188556, PMID: 33932560 PMC8730328

[74] Ruan J Wu Y Wang H Huang Z Liu Z Yang X. Graph theory analysis of a human body metabolic network: A systematic and organ-specific study. Med Phys. (2025) 52:2340–55. doi: 10.1002/mp.17568, PMID: 39680791

[75] Castellanos-Rueda R Di Roberto RB Bieberich F Schlatter FS Palianina D Nguyen OTP. speedingCARs: accelerating the engineering of CAR T cells by signaling domain shuffling and single-cell sequencing. Nat Commun. (2022) 13:6555. doi: 10.1038/s41467-022-34141-8, PMID: 36323661 PMC9630321

[76] Pandey N Biswas D Dutta N Hansda A Dutta G Mukherjee G. Sensing soluble immune checkpoint molecules and disease-relevant cytokines in cancer: A novel paradigm in disease diagnosis and monitoring. Front Sens. (2022) 3:789771. doi: 10.3389/fsens.2022.789771

[77] Fenton KA Pedersen HL. Advanced methods and novel biomarkers in autoimmune diseases − a review of the recent years progress in systemic lupus erythematosus. Front Med. (2023) 10:1183535. doi: 10.3389/fmed.2023.1183535, PMID: 37425332 PMC10326284

[78] Keresztes D Kerestély M Szarka L Kovács BM Schulc K Veres DV. Cancer drug resistance as learning of signaling networks. BioMed Pharmacother. (2025) 183:117880. doi: 10.1016/j.biopha.2025.117880, PMID: 39884030

[79] Nair A Chauhan P Saha B Kubatzky KF. Conceptual evolution of cell signaling. Int J Mol Sci. (2019) 20:3292. doi: 10.3390/ijms20133292, PMID: 31277491 PMC6651758

[80] Burns MS Miramontes R Wu J Gulia R Saddala MS Lau AL. Distinct molecular patterns in R6/2 HD mouse brain: Insights from spatiotemporal transcriptomics. Neuron. (2025). doi: 10.1016/j.neuron.2025.05.014, PMID: 40482637 PMC12279385

[81] Ramezani M Weisbart E Bauman J Singh A Yong J Lozada M. A genome-wide atlas of human cell morphology. Nat Methods. (2025) 22:621–33. doi: 10.1038/s41592-024-02537-7, PMID: 39870862 PMC11903339

[82] SenGupta S Parent CA Bear JE. The principles of directed cell migration. Nat Rev Mol Cell Biol. (2021) 22:529–47. doi: 10.1038/s41580-021-00366-6, PMID: 33990789 PMC8663916

[83] De la Fuente IM Martínez L Carrasco-Pujante J Fedetz M López JI Malaina I. Self-organization and information processing: from basic enzymatic activities to complex adaptive cellular behavior. Front Genet. (2021) 12:644615. doi: 10.3389/fgene.2021.644615, PMID: 34093645 PMC8176287

[84] Agliari E Annibale A Barra A Coolen ACC Tantari D. Immune networks: multi-tasking capabilities at medium load. (2013). doi: 10.48550/arXiv.1302.7259

[85] Céspedes PF Beckers D Dustin ML Sezgin E. Model membrane systems to reconstitute immune cell signaling. FEBS J. (2021) 288:1070–90. doi: 10.1111/febs.15488, PMID: 32681663

[86] Shalizi CR. Methods and techniques of complex systems science: an overview. In: Deisboeck TS Kresh JY, editors. Complex Systems Science in Biomedicine. Springer US, Boston, MA (2006). p. 33–114. doi: 10.1007/978-0-387-33532-2_2

[87] Santamaria K Desmots F Leonard S Caron G Haas M Delaloy C. Committed human CD23-negative light-zone germinal center B cells delineate transcriptional program supporting plasma cell differentiation. Front Immunol. (2021) 12:744573. doi: 10.3389/fimmu.2021.744573, PMID: 34925321 PMC8674954

[88] Zheng Z. An introduction to emergence dynamics in complex systems. In: Liu X-Y, editor. Frontiers and Progress of Current Soft Matter Research. Springer, Singapore (2021). p. 133–96. doi: 10.1007/978-981-15-9297-3_4

[89] Berumen Sánchez G Bunn KE Pua HH Rafat M. Extracellular vesicles: mediators of intercellular communication in tissue injury and disease. Cell Commun Signal. (2021) 19:104. doi: 10.1186/s12964-021-00787-y, PMID: 34656117 PMC8520651

[90] Artinger M Gerken OJ Purvanov V Legler DF. Distinct fates of chemokine and surrogate molecule gradients: consequences for CCR7-guided dendritic cell migration. Front Immunol. (2022) 13:913366. doi: 10.3389/fimmu.2022.913366, PMID: 35769489 PMC9234131

[91] Garg AK Mitra T Schips M Bandyopadhyay A Meyer-Hermann M. Amount of antigen, T follicular helper cells and affinity of founder cells shape the diversity of germinal center B cells: A computational study. Front Immunol. (2023) 14:1080853. doi: 10.3389/fimmu.2023.1080853, PMID: 36993964 PMC10042134

[92] Erenpreisa J Giuliani A Yoshikawa K Falk M Hildenbrand G Salmina K. Spatial-temporal genome regulation in stress-response and cell-fate change. Int J Mol Sci. (2023) 24:2658. doi: 10.3390/ijms24032658, PMID: 36769000 PMC9917235

[93] Huang Q Li Y Huang Y Wu J Bao W Xue C. Advances in molecular pathology and therapy of non-small cell lung cancer. Signal Transduct Target Ther. (2025) 10:186. doi: 10.1038/s41392-025-02243-6, PMID: 40517166 PMC12167388

[94] Netea MG Schlitzer A Placek K Joosten LAB Schultze JL. Innate and adaptive immune memory: an evolutionary continuum in the host’s response to pathogens. Cell Host Microbe. (2019) 25:13–26. doi: 10.1016/j.chom.2018.12.006, PMID: 30629914

[95] Adams NM Grassmann S Sun JC. Clonal expansion of innate and adaptive lymphocytes. Nat Rev Immunol. (2020) 20:694–707. doi: 10.1038/s41577-020-0307-4, PMID: 32424244 PMC13119617

[96] Tan P He L Huang Y Zhou Y. Optophysiology: Illuminating cell physiology with optogenetics. Physiol Rev. (2022) 102:1263–325. doi: 10.1152/physrev.00021.2021, PMID: 35072525 PMC8993538

[97] Aghamiri SS Puniya BL Amin R Helikar T. A multiscale mechanistic model of human dendritic cells for in-silico investigation of immune responses and novel therapeutics discovery. Front Immunol. (2023) 14:1112985. doi: 10.3389/fimmu.2023.1112985, PMID: 36993954 PMC10040975

[98] Hunter P. Understanding redundancy and resilience. EMBO Rep. (2022) 23:e54742. doi: 10.15252/embr.202254742, PMID: 35156768 PMC8892264

[99] Charles A Janeway J Travers P Walport M Shlomchik MJ. The importance of immunological memory in fixing adaptive immunity in the genome. In: Immunobiology: The Immune System in Health and Disease. 5th edition. Garland Science: Garland Science (2001). Available online at: https://www.ncbi.nlm.nih.gov/books/NBK27087/.

[100] Mariani E Lisignoli G Borzì RM Pulsatelli L. Biomaterials: foreign bodies or tuners for the immune response? Int J Mol Sci. (2019) 20:636. doi: 10.3390/ijms20030636, PMID: 30717232 PMC6386828

[101] Millar-Wilson A Ward Ó Duffy E Hardiman G. Multiscale modeling in the framework of biological systems and its potential for spaceflight biology studies. iScience. (2022) 25:105421. doi: 10.1016/j.isci.2022.105421, PMID: 36388986 PMC9663911

[102] Widhalm D Goeschka KM Kastner W. A review on immune-inspired node fault detection in wireless sensor networks with a focus on the danger theory. Sensors. (2023) 23:1166. doi: 10.3390/s23031166, PMID: 36772205 PMC9920811

[103] Altan-Bonnet G Mukherjee R. Cytokine-mediated communications: a quantitative appraisal of immune complexity. Nat Rev Immunol. (2019) 19:205–17. doi: 10.1038/s41577-019-0131-x, PMID: 30770905 PMC8126146

[104] Ordovas-Montanes J Beyaz S Rakoff-Nahoum S Shalek AK. Distribution and storage of inflammatory memory in barrier tissues. Nat Rev Immunol. (2020) 20:308–20. doi: 10.1038/s41577-019-0263-z, PMID: 32015472 PMC7547402

[105] Chu C Artis D Chiu IM. Neuro-immune interactions in the tissues. Immunity. (2020) 52:464–74. doi: 10.1016/j.immuni.2020.02.017, PMID: 32187517 PMC10710744

[106] Ramot A Jiang Z Tian J-B Nahum T Kuperman Y Justice N. Hypothalamic CRFR1 is essential for HPA axis regulation following chronic stress. Nat Neurosci. (2017) 20:385–8. doi: 10.1038/nn.4491, PMID: 28135239

[107] Rowland I Gibson G Heinken A Scott K Swann J Thiele I. Gut microbiota functions: metabolism of nutrients and other food components. Eur J Nutr. (2018) 57:1–24. doi: 10.1007/s00394-017-1445-8, PMID: 28393285 PMC5847071

[108] Boahen A Hu D Adams MJ Nicholls PK Greene WK Ma B. Bidirectional crosstalk between the peripheral nervous system and lymphoid tissues/organs. Front Immunol. (2023) 14:1254054. doi: 10.3389/fimmu.2023.1254054, PMID: 37767094 PMC10520967

[109] Leysen H Walter D Christiaenssen B Vandoren R Harputluoğlu İ Van Loon N. GPCRs are optimal regulators of complex biological systems and orchestrate the interface between health and disease. Int J Mol Sci. (2021) 22:13387. doi: 10.3390/ijms222413387, PMID: 34948182 PMC8708147

[110] Tsigos C Kyrou I Kassi E Chrousos GP. Stress: endocrine physiology and pathophysiology. In: Feingold KR Ahmed SF Anawalt B Blackman MR Boyce A Chrousos G, editors. Endotext. MDText.com, Inc, South Dartmouth (MA (2000). Available online at: http://www.ncbi.nlm.nih.gov/books/NBK278995/.

[111] Hartsock MJ Strnad HK Spencer RL. Iterative metaplasticity across timescales: how circadian, ultradian, and infradian rhythms modulate memory mechanisms. J Biol Rhythms. (2022) 37:29–42. doi: 10.1177/07487304211058256, PMID: 34781753 PMC9236757

[112] Liu Y Yu Y Liu J Liu W Cao Y Yan R. Neuroimmune regulation in sepsis-associated encephalopathy: the interaction between the brain and peripheral immunity. Front Neurol. (2022) 13:892480. doi: 10.3389/fneur.2022.892480, PMID: 35832175 PMC9271799

[113] Jiang M Wang Y Zhao X Yu J. From metabolic byproduct to immune modulator: the role of lactate in tumor immune escape. Front Immunol. (2024) 15:1492050. doi: 10.3389/fimmu.2024.1492050, PMID: 39654883 PMC11625744

[114] Daëron M. The immune system as a system of relations. Front Immunol. (2022) 13:984678. doi: 10.3389/fimmu.2022.984678, PMID: 36177051 PMC9513551

[115] Petrova B Guler AT. Recent developments in single-cell metabolomics by mass spectrometry─A perspective. J Proteome Res. (2025) 24:1493–518. doi: 10.1021/acs.jproteome.4c00646, PMID: 39437423 PMC11976873

[116] Cohen IR Efroni S. The immune system computes the state of the body: crowd wisdom, machine learning, and immune cell reference repertoires help manage inflammation. Front Immunol. (2019) 10:10. doi: 10.3389/fimmu.2019.00010, PMID: 30723470 PMC6349705

[117] Megha KB Joseph X Akhil V Mohanan PV. Cascade of immune mechanism and consequences of inflammatory disorders. Phytomedicine. (2021) 91:153712. doi: 10.1016/j.phymed.2021.153712, PMID: 34511264 PMC8373857

[118] Vishwanathan A Sood A Wu J Ramirez AD Yang R Kemnitz N. Predicting modular functions and neural coding of behavior from a synaptic wiring diagram. Nat Neurosci. (2024) 27:2443–54. doi: 10.1038/s41593-024-01784-3, PMID: 39578573 PMC11614741

[119] Khona M Chandra S Fiete I. Emergence of robust global modules from local interactions and smooth gradients. (2024). doi: 10.1101/2021.10.28.466284 39972140

[120] Buckner RL Sepulcre J Talukdar T Krienen FM Liu H Hedden T. Cortical hubs revealed by intrinsic functional connectivity: mapping, assessment of stability, and relation to Alzheimer’s disease. J Neurosci. (2009) 29:1860–73. doi: 10.1523/JNEUROSCI.5062-08.2009, PMID: 19211893 PMC2750039

[121] Kaviani S Sohn I. Application of complex systems topologies in artificial neural networks optimization: An overview. Expert Syst Appl. (2021) 180:115073. doi: 10.1016/j.eswa.2021.115073

[122] Efatmaneshnik M Reidsema C. Immunity as a design decision making paradigm for complex systems: A robustness approach. Cybern Syst. (2007) 38:759–80. doi: 10.1080/01969720701601056

[123] Axenie C López-Corona O Makridis MA Akbarzadeh M Saveriano M Stancu A. Antifragility as a complex system’s response to perturbations, volatility, and time. Taylor & Francis: ArXiv (2023). Available online at: https://www.ncbi.nlm.nih.gov/pmc/articles/PMC10775345/. arXiv:2312.13991v1.

[124] Agrawal B. Heterologous immunity: role in natural and vaccine-induced resistance to infections. Front Immunol. (2019) 10:2631. doi: 10.3389/fimmu.2019.02631, PMID: 31781118 PMC6856678

[125] Tokalić R Viđak M Kaknjo MM Marušić A. Antifragility of healthcare systems in Croatia and Bosnia and Herzegovina: Learning from man-made and natural crises. Lancet Reg Health Eur. (2021) 9:100216. doi: 10.1016/j.lanepe.2021.100216, PMID: 34693390 PMC8513139

[126] Minati G. Theoretical reflections on reductionism and systemic research issues: dark systems and systemic domains. Systems. (2024) 12:2. doi: 10.3390/systems12010002

[127] Corti A Colombo M Migliavacca F Rodriguez Matas JF Casarin S Chiastra C. Multiscale computational modeling of vascular adaptation: A systems biology approach using agent-based models. Front Bioeng Biotechnol. (2021) 9:744560. doi: 10.3389/fbioe.2021.744560, PMID: 34796166 PMC8593007

[128] Niarakis A Laubenbacher R An G Ilan Y Fisher J Flobak Å. Immune digital twins for complex human pathologies: applications, limitations, and challenges. NPJ Syst Biol Appl. (2024) 10:141. doi: 10.1038/s41540-024-00450-5, PMID: 39616158 PMC11608242

[129] Kraus A Buckley KM Salinas I. Sensing the world and its dangers: An evolutionary perspective in neuroimmunology. eLife. (2024) 10:e66706. doi: 10.7554/eLife.66706, PMID: 33900197 PMC8075586

[130] Brouillet MJ Georgiev GY. Why and How do Complex Systems Self-Organize at All? Average Action Efficiency as a Predictor, Measure, Driver, and Mechanism of Self-Organization. Processes. (2024) 12:2937. doi: 10.3390/pr12122937

[131] Xu X Su J Zhu R Li K Zhao X Fan J. From morphology to single-cell molecules: high-resolution 3D histology in biomedicine. Mol Cancer. (2025) 24:63. doi: 10.1186/s12943-025-02240-x, PMID: 40033282 PMC11874780

[132] Sacchi S Malagoli D Franchi N. The invertebrate immunocyte: A complex and versatile model for immunological, developmental, and environmental research. Cells. (2024) 13:2106. doi: 10.3390/cells13242106, PMID: 39768196 PMC11674123

[133] Sadhu T. Emergence and complexity in theoretical models of self-organized criticality. (2017). doi: 10.48550/arXiv.1701.01125

[134] De Domenico M Allegri L Caldarelli G d’Andrea V Di Camillo B Rocha LM. Challenges and opportunities for digital twins in precision medicine from a complex systems perspective. NPJ Digit Med. (2025) 8:37. doi: 10.1038/s41746-024-01402-3, PMID: 39825012 PMC11742446

